# The prevalence and associated factors for delayed presentation for HIV care among tuberculosis/HIV co-infected patients in Southwest Ethiopia: a retrospective observational cohort

**DOI:** 10.1186/s40249-016-0193-y

**Published:** 2016-11-02

**Authors:** Hailay Gesesew, Birtukan Tsehaineh, Desalegn Massa, Amanuel Tesfay, Hafte Kahsay, Lillian Mwanri

**Affiliations:** 1 Department of Epidemiology, College of Health Sciences, Jimma University, Jimma, Ethiopia; 2 Discipline of Public Health, Faculty of Medicine, Nursing and Health Sciences, Flinders University, Adelaide, Australia; 3 School of Statistics and Mathematics, Faculty of Science, Alberta University, Edmonton, Canada; 4 Department of population and Family Health, College of Health Sciences, Jimma University, Jimma, Ethiopia; 5 ART Clinic, Filtu Hospital, Somali, Ethiopia

**Keywords:** Tuberculosis, HIV, Co-infection, Delayed presentation, Late presentation, Retrospective cohort, Prevalence, Ethiopia

## Abstract

**Background:**

A delay presentation for human immunodeficiency virus (HIV) patient’s care (that is late engagement to HIV care due to delayed HIV testing or delayed linkage for HIV care after the diagnosis of HIV positive) is a critical step in the series of HIV patient care continuum. In Ethiopia, delayed presentation (DP) for HIV care among vulnerable groups such as tuberculosis (Tb) /HIV co-infected patients has not been assessed. We aimed to assess the prevalence of and factors associated with DP (CD4 < 200 cells/μl at first visit) among Tb/HIV co-infected patients in southwest Ethiopia.

**Methods:**

A retrospective observational cohort study collated Tb/HIV data from Jimma University Teaching Hospital for the period of September 2010 and August 2012. The data analysis used logistic regression model at *P* value of ≤ 0.05 in the final model.

**Results:**

The prevalence of DP among Tb/HIV co-infected patients was 59.9 %. Tb/HIV co-infected patients who had a house with at least two rooms were less likely (A*OR*, 0.5; 95 % *CI*: 0.3–1.0) to present late than those having only single room. Tobacco non-users of Tb/HIV co-infected participants were also 50 % less likely (A*OR*, 0.5; 95 % *CI*: 0.3–0.8) to present late for HIV care compared to tobacco users. The relative odds of DP among Tb/HIV co-infected patients with ambulatory (A*OR*, 1.8; 95 % *CI*, 1.0–3.1) and bedridden (A*OR*, 8.3; 95 % *CI*, 2.8–25.1) functional status was higher than with working status.

**Conclusions:**

Three out of five Tb/HIV co-infected patients presented late for HIV care. Higher proportions of DP were observed in bedridden patients, tobacco smokers, and those who had a single room residence. These findings have intervention implications and call for effective management strategies for Tb/HIV co-infection including early HIV diagnosis and early linkage to HIV care services.

**Electronic supplementary material:**

The online version of this article (doi:10.1186/s40249-016-0193-y) contains supplementary material, which is available to authorized users.

## Multilingual abstract

Please see Additional file 1 for translations of the abstract into the six official working languages of the United Nations.

## Background

HIV care continuum is a series of steps from the time a person is diagnosed with HIV through assessment for antiretroviral therapy (ART) eligibility, retention in care, and immunologic success and virologic suppression via treatment adherence [1]. A myriad of activities have been devoted to mitigate negative HIV outcomes in the continuum [2]. Nevertheless, challenges exist at every step of the continuum. Timely HIV testing is the first critical step in effective HIV care and prevention [3]. HIV infected persons fail to get tested due to various factors. Thus include: being unaware of their risk of contracting infection or importance of getting oneself tested and not being able to access care promptly once they test positive [4, 5]. Hence, delayed presentation for HIV care (DP) can either be due to delay in HIV testing or delay in linkage with or in accessing the HIV care.

There is little consensus on what should be considered DP, and several definitions have been used to date. Some have defined DP when the diagnosis of an AIDS defining condition occurs either before or concomitantly to an HIV diagnosis [6], during the subsequent six months [5, 7] or during the following year of an HIV diagnosis [8]. Other definitions of DP use CD4 cell count of < 200 [9] or < 350 [10] cells/μl. The 1993 expanded AIDS surveillance case definition measured DP if persons present with a CD4 cell count < 200 cells/μl and/or with an AIDS defining disease [11].

DP has several consequences including: (i) increased risk of progression of the infection; ii) increased risk of HIV transmission with severe public health implications [12]; iii) facilitation of immunological failure and treatment failure [13–15]; (iv) increased risk of poor treatment outcomes including early mortality [13–16]; and v) increased first line ART drug resistance due to multiplication and then mutation of the virus, and thereby switching to more expensive second line regimens [12]. In addition, DP also challenges the effectiveness of test-and-treat strategies [17]. Test-and-treat strategies for HIV care theorize that earlier testing and treatment of HIV infection could switch prominently with significantly ongoing HIV transmission and further curtail the HIV epidemic [17].

DP has been reported to be a significant problem across the world in developed and developing countries. In Europe for example, the prevalence of DP has been reported to be roughly between 15 and 66 % [18, 19]. Higher prevalence of 72–83.3 % [20, 21] has been reported from Asia. In Africa, 35–65 % report late for HIV care [22–25]. Reported barriers to DP among general population have include several factors including: age, sex, level of education, income, place of residence, perceiving HIV as curable, HIV related stigma, co-morbidity, having contact with female sex workers, alcohol users, chewing chat, smoking cigarette, perceived risky sexual behaviour, pre-and post-test counseling [4, 15, 20, 21, 26, 27].

While there have been no appropriate studies to estimate the prevalence of DP in Ethiopia, a situational analysis conducted in southwest part of the country reported that 33.1 % of patients from a health center and 38.4 % of patients from a hospital presented late for the care [28]. A few studies have assessed reasons for DP among the general HIV population [4, 15, 27]. However, there is a lack of studies that have explored DP among vulnerable groups such as tuberculosis (Tb) /HIV co-infected patients.

Tb and HIV, the most important infectious diseases of our era, are inextricably linked [29]. HIV-1 and *Mycobacterium tuberculosis* (M.Tb) are both intracellular pathogens having a potential to interact at different levels such as population, clinical, and cellular [30]. Their co-infection causes serious bidirectional effect than one causes alone [30]. Both cause synergistic combination of illness in which HIV promotes the progression of latent Tb infection to disease, and Tb accelerates the progression of HIV disease to poor prognosis including death [31]. In addition, HIV has been ascribed as the principal factor for failure to meet Tb control targets in HIV endemic settings, and Tb is a key cause of death among people living with HIV in similar settings [32]. Given the above facts, DP among Tb/HIV co-infected patients should be given a top priority in order to curb both scourges. This study aimed to assess the prevalence of and factors associated with DP among Tb/HIV co-infected patients.

## Methods

### Design and population

A retrospective observational cohort study was undertaken between August and October 2013 using records from September 01, 2010 to August 31 2012 in ART clinic at Jimma University Teaching Hospital (JUTH). JUTH is situated in Jimma zone, 357 km southwest of Addis Ababa, the capital city of Ethiopia. The zone has an estimated population of 2 486 155 people of which 89.69 % are rural inhabitants [33].

Jimma is found in a regional state (Oromia) that accounted for the highest number of HIV infected people from Ethiopia with 224 000 people. It is near Gambella region, a regional state that had the highest prevalence rate of HIV from Ethiopia with 6 % [34]. There is a large refugee camp near Jimma. Refugees from many African countries pour into this camp. The movement of these people to and from the city increases the risk of HIV infection and Tb in both the city and the camp. In Jimma, primary healthcare services, including diagnosis and treatment of Tb, voluntary counseling and testing (VCT), prevention of mother to child transmission (PMTCT), ART and opportunistic infections (OIs) treatment services are available. All patients aged ≥15 years and who had access to Tb/HIV medical treatment at JUTH were the target population.

### Data source

JUTH has an electronic patient database called Comprehensive Care Centre Patient Application Database (C-PAD). C-PAD is Electronic Medical Records or EMR system database that contains patients’ both clinical and non-clinical information. This was the main source of data in this study. Data were extracted using a data extraction check list from the database. Data clerks immediately informed the clinicians if any data was missing, and weekly EMR-generated patient summary reports help to flag patients with conditions that needed follow-up. When data were incomplete, we tried to refer the patients’ cards, registration and log books.

### Study variables

Delayed presentation for HIV care is the response variable and was dichotomized as delayed and early. DP refers to HIV positive individuals aged 15 years and above having the CD4 lymphocyte count of less than 200/μl irrespective of clinical staging at the time of first presentation to the ART clinics of the institution. Early presentation for HIV care refers to HIV positive individuals aged 15 years and above having the CD4 lymphocyte count of ≥ 200/μl irrespective of clinical staging at the time of first presentation to the ART clinics of the institution.

The explanatory variables included age, sex, educational level, marital status, occupation, residence, number of people living in the household, accessibility to safe water, accessibility of electricity, number of bedrooms in the household, functional status, disclosure, condom use, risky sexual behavior, tobacco smoking, alcohol drinking, Tb type and mode of entry. Level of education was classified as illiterate (could not read and write), read and write only (could read and write but had received no formal education) and formal education (received formal education starting from grade one). Functional status was categorized as work (able to perform usual work), ambulatory (able to perform activity of daily living) and bedridden (not able to perform activity of daily living). Mode of entry was the mode of anti-Tb treatment entry of patients and was categorized as new, relapse and dropout.

### Data analyses

Data exploration, editing and cleaning were undertaken before analysis. The analysis of both descriptive and inferential statistics was conducted. Descriptive statistics included mean and standard deviation values for continuous data; percentage and frequency tables for categorical data. Logistic regression was used to identify factors associated with DP. Bivariate logistic regression analysis was conducted to see the existence of crude association and select candidate variables (with *P* value below 0.25) to multiple logistic regression. We checked multi-collinearity among selected independent variables via variance inflation factor (VIF) and none was found. *P*-value ≤ 0.05 was considered as a cut point for statistical significance in the final model. Fitness of goodness of the final model was checked by Hosmer and Lemeshow test and was found fit. The data was summarized using odds ratio (OR) and 95 % confidence interval. Data analysis was conducted using SPSS version 21 for mackintosh.

## Results

### Demographic characteristics of study participants

**Fig. 1:**
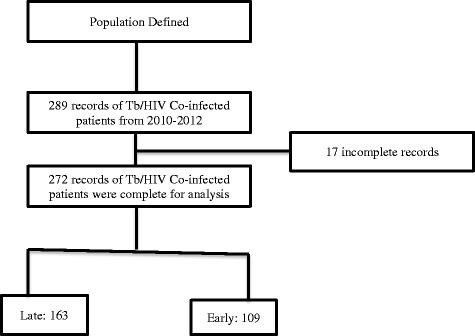
Schematic presentation of data extraction of delayed presentation for HIV care among Tb/HIV co-infected patients

Two hundred and eighty nine (289) Tb/HIV co-infected patients were registered for HIV care during the period between September 2010 and August 2012 in JUTH (Fig. 1), but 17 records were incomplete in all data sources. Table 1 shows demographic characteristics of the 272 Tb/HIV co-infected respondents. The majority of the study participants were between 25 and 34 years with a mean age of 32 (±8.53) years, and females accounted for more than half (58.1 %) of the study participants. About half (51.4 %) of the respondents followed Muslim religion and one third (31.6 %) of participants represented daily laborers. More than half of the population (51.5 %) had formal education and two third (60.7 %) of the respondents were married. Urban dwellers were over-represented (70 %).

**Table 1:** Demographic characteristics of Tb/HIV co-infected patients at JUTH, Southwest Ethiopia, 2013

Variable	Category	Number (*n* = 272)	Percent
Age (in years)	15–24	38	13.9
	25–34	140	51.5
	35–44	69	25.4
	≥ 45	25	9.2
	Mean age (± SD)	32 (±8.53) years	
Sex	Male	114	41.9
	Female	158	58.1
Occupation	Government employed	48	17.7
	NGO	46	16.9
	Farmer	80	29.4
	Daily labor	86	31.6
	Female Sex Worker	12	4.4
Educational status	Illiterate	81	29.8
	Read & write (informal)	51	18.8
	Formal education	140	51.4
Religion	Orthodox	76	27.9
	Muslim	134	49.3
	Protestant	50	18.4
	Catholic	12	4.4
Marital status	Married	165	60.7
	Single	58	21.3
	Divorced	36	13.2
	Widowed	13	4.8
Residence	Urban	189	69.5
	Rural	83	30.5

*Tb/HIV* tuberculosis/human immunodeficiency virus, *JUTH* Jimma University Teaching Hospital, *NGO* Non Governmental Organization, *SD* standard deviation

### Prevalence of delayed presentation for HIV care and characteristics of delayed presenters

A total of 163 (59.9 %) Tb/HIV co-infected patients were categorised as delayed presenters for HIV care during the study period. Table 2 presents demographic and clinical characteristics of DP. Tb/HIV participants aged between 25 and 34 years, and 35 and 44 years contributed for 46.6 % and 28.2 % of DP proportion respectively. Females accounted for more than half (55.8 %) of DP. DP was also higher among married (61.3 %) compared to single (20.9 %) study participants. DP was very high among occupants with economic hardship. Farmers and daily laborers formed 32.5 and 27 % of DP amongst the study participants respectively. When analyzed by level of education, about half (48.5 %) of the participants who presented late for HIV care were formally educated whereas one third (30.1 %) were illiterate. The remaining delayed presenters were noted as being able to read and write but did not have formal education. Two third (66.9 %) of delayed presenters were urban dwellers.

**Table 2:** Demographic, clinical and behavioral characteristics of Tb/HIV co-infected patients by time to presentation for HIV care at JUTH, Southwest Ethiopia, 2013

Variable	Category	Time to present for HIV care		C*OR* (95 % *CI*)
		Early, *n* (%)	Late, *n* (%)	
Age^a^	< 25 years	15 (13.8)	23 (14.1)	1
	25–34 years	64 (58.7)	76 (46.6)	0.8 (0.4–1.6)
	35–44 years	23 (21.1)	46 (28.2)	1.3 (0.6–2.9)
	≥ 45 years	7 (6.4)	18 (11.1)	1.7 (0.6–4.9)
Sex	Male	42 (38.5)	72 (44.2)	1
	Female	67 (61.5)	91 (55.8)	0.8 (0.5–1.3)
Marital status	Single	24 (22)	34 (20.9)	1
	Married	65 (59.6)	100 (61.3)	1.1 (0.6–2.0)
	Divorced	13 (11.9)	23 (14.1)	1.3 (0.5–2.9)
	Widowed	7 (6.4)	6 (3.7)	0.6 (0.2–2.0)
Education	Illiterate	32 (29.4)	49 (30.1)	1
	Read and Write	16 (14.7)	35 (21.5)	1.4 (0.7–3.0)
	Formal Education	61 (56)	79 (48.5)	0.9 (0.5–1.5)
Occupation^a^	Government Employee	21 (19.3)	27 (16.6)	1
	NGO	14 (12.8)	32 (19.6)	1.8 (0.8–4.2)
	Farmer	27 (24.8)	53 (32.5)	1.5 (0.8–3.2)
	Daily Labor	42 (38.5)	44 (27)	0.8 (0.4–1.6)
	FSW	5 (4.6)	7 (4.3)	1.1 (0.3–3.9)
Residence^a^	Urban	80 (73.4)	109 (66.9)	1
	Rural	29 (26.6)	54 (33.1)	1.4 (0.8–2.3)
Number of rooms^a^	1	44 (40.4)	77 (47.2)	1
	2	55 (50.5)	55 (33.7)	0.6 (0.3–0.9)
	3	8 (7.3)	23 (14.1)	1.6 (0.7–3.9)
	4	2 (1.8)	8 (4.9)	2.3 (0.5–11.2)
Water^a,b^	Yes	77 (70.6)	130 (79.8)	1
	No	32 (29.4)	33 (20.2)	0.6 (0.4–1.1)
Electricity	Yes	79 (72.5)	128 (78.5)	1
	No	30 (27.5)	35 (21.5)	0.7 (0.4–1.3)
Disclosure	Yes	67 (61.5)	109 (66.9)	1
	No	42 (38.5)	54 (33.1)	1.3 (0.8–2.1)
Partner’s HIV Status	Positive	72 (66.1)	117 (71.8)	1
	Negative/Unknown	37 (33.9)	46 (28.2)	0.8 (0.5–1.3)
Risky Behavior	Yes	78 (71.6)	113 (69.3)	1
	No	31 (28.4)	50 (30.7)	1.1 (0.7–1.9)
Condom Use	Yes	14 (12.8)	33 (20.2)	1
	No	95 (87.2)	130 (79.8)	0.6 (0.3–1.2)
Tobacco use^a^	Yes	40 (36.7)	86 (52.8)	1
	No	69 (63.3)	77 (47.2)	0.5 (0.3–0.9)
Drinking alcohol	Yes	29 (26.6)	47 (28.8)	1
	No	(80 73.4)	116 (71.2)	0.9 (0.5–1.5)
Functional status^a^	Work	68 (62.4)	70 (42.9)	1
	Ambulatory	37 (33.9)	58 (35.6)	1.5 (0.9–2.6)
	Bedridden	4 (3.7)	35 (21.5)	8.5 (2.9–25.2)
Tb Type	Pulmonary	90 (82.6)	123 (75.5)	1
	Extra Pulmonary	5 (4.6)	9 (5.5)	1.3 (0.4–4.1)
	Mixed	14 (12.8)	31 (19)	1.6 (0.8–3.2)
Mode of Tb treatment Entry	New	91 (83.5)	141 (86.5)	1
	Relapse	11 (10.1)	16 (9.8)	0.9 (0.4–2.1)
	Dropout	7 (6.4)	6 (3.7)	0.6 (0.2–1.7)

*HIV* human immunodeficiency virus, *JUTH* Jimma University Teaching Hospital, *Tb* tuberculosis, *FSW* female sex workers

^a^Candidate variables for multiple logistic regression (*P*-value < 0.25)

^b^Availability of safe water

Among the delayed presenters, 77 (47.2) and 55 (33.7 %) study participants had houses with single and double bedrooms respectively; whereas households with four bedrooms accounted for only 4.9 % of delayed presenters. A fifth, i.e. 20.2 and 21.5 % of delayed presenters did not have water and electricity in their households. An additional 21.5 % of delayed presenters were seriously ill and bedridden. The majority (75.5 %) of delayed presenters had pulmonary Tb type followed by mixed type (19 %). Tb/HIV participants who were exposed to risky behaviors such as having multiple sexual partners accounted for 69.3 % of DP.

### Factors associated with delayed presentation for HIV care

Age, occupational status, place of residence, number of rooms per household, availability of safe water, smoking tobacco and functional status had *P*-value ≤ 0.25 in bivariate logistic regression and were candidates for multiple logistic regression (Table 2).

Table 3 presents the multiple logistic regression analysis with DP. Logistic regression analyses demonstrated the following were associated with DP: number of rooms per household, being recorded as tobacco user and being recorded as ambulatory or bedridden functional status. Tb/HIV co-infected patients who had houses with double rooms were less likely (A*OR*, 0.5; 95 % *CI*: 0.3–1.0) to present late than those having only single room. Tobacco non-users of Tb/HIV co-infected participants were also 50 % less likely (A*OR*, 0.5; 95 % *CI*: 0.3–0.8) to present late for HIV care compared to tobacco users. The relative odds of DP among patients with working status was lower compared to patients with bedridden (A*OR*, 8.3; 95 % *CI*, 2.8–25.1) and ambulatory (A*OR*, 1.8; 95 % *CI*, 1.0–3.1) status.

**Table 3:** Multiple logistic regression predictors of delayed presentation for HIV care at JUTH, Southwest Ethiopia, 2013

Variable	Category	Time to present for HIV care (*n* = 272)		Odds Ratio	
		Early, *n* (%)	Late, *n* (%)	Crude *OR* (95%*CI*)	Adjusted *OR* (95 % *CI*)
Number of rooms	1	44 (40.4)	77 (47.2)	1	1
	2	55 (50.5)	55 (33.7)	0.6 (0.3–0.9)	0.5 (0.3–1.0)*
	3	8 (7.3)	23 (14.1)	1.6 (0.7–3.9)	1.7 (0.7–4.4)
	4	2 (1.8)	8 (4.9)	2.3 (0.5–11.2)	1.7 (0.3–8.9)
Tobacco use	Yes	154 (81.5)	35 (18.5)	1	1
	No	63 (75.9)	20 (24.1)	1.4 (0.8–2.6)	0.5 (0.3–0.8)*
Functional status	Work	68 (62.4)	70 (42.9)	1	1
	Ambulatory	37 (33.9)	58 (35.6)	1.8 (1.0–3.1)	1.8 (1.0–3.1)*
	Bedridden	4 (3.7)	35 (21.5)	2.2 (0.9–4.7)	8.3 (2.8–25.1)*

*JUTH* Jimma University Teaching Hospital*, OR* odds ratio, *CI* confidence interval

*Denotes statistically significant in final model at *P* value of ≤ 0.05 in the final model

## Discussion

The UNAIDS 90-90-90 goal for 2020 aims to of diagnose 90 % of people living with HIV, provide antiretroviral therapy (ART) to 90 % of those diagnosed and achieve 90 % viral load suppression among those on treatment [35]. The trend of expansion of HIV care services particularly ART treatment in Ethiopia is promising. ART program was expanded from four facilities in 2003 to 913 in 2013, and the number of people on ART has increased from 900 at the beginning of 2005 to 270,460 in 2012 [36, 37]. However, not much attention has been given to prevent DP for the vulnerable groups particularly Tb/HIV co-infected patients which remains one of the vastest defies in the reduction of HIV infection in resource scare countries including Ethiopia. Nearly 60 % of the patients in our study were delayed presenters, a finding that is similar with a study conducted in Zimbabwe [38]. This is also a comparable magnitude with DP of general HIV population in Africa [22, 26].

However, in Ethiopia, the prevalence of DP among TB/HIV co-infected patients according to the current study is about twice as compared to the previous finding conducted among general HIV population [4]. This indicates that the prevalence of DP among Tb/HIV co-infected patients is a considerable number. This is against the current treatment guidelines of WHO and Ethiopia that advocate early commencement of ART among Tb/HIV co-infected patients [32, 39, 40]. Such an impediment to engage for HIV care poses a sizeable obstacle to the successful implementation of strategies that suggest “test” (i.e. early identification of all HIV-infected individuals) and “treat” (i.e. initiation of antiretroviral therapy in these individuals) [41–43]. Previous studies confirmed that “test” and “treat” strategy could have dramatic reductions in the incidence of HIV infection and transmission [41–43].

The timing of ART treatment initiation among Tb/HIV co-infected patients is critically important for the favorable therapeutic outcomes and patient care [39]. According to the current guidelines, ART should be started within 2–8 weeks after commencement of anti-Tb treatment [44]. Nevertheless, the issue of when to start ART in Tb patients has been debatable [45, 46]. Early or concurrent starting of ART may lead to high pill burden, clinical debilitation due to immune reconstitution inflammatory syndrome (IRIS), toxicity of drugs, decrease drug compliance, worsening of ailment, and finally demise [45, 46]. To the contrary, late commencement of ART may lead to exacerbation of ailment and death [45, 46].

Findings of a previous study conducted in Zimbabwe [38] depicted that being treated for Tb first time, staying more than 5 km from a clinic, and having a family member on ART were factors for delayed ART initiation. In addition, findings of another study from Malawi [47] revealed that cost of transport to the hospital ART site was significantly associated with ART acceptance. This indicates that ART acceptance among Tb patients in a rural district in Malawi is low and may engage to care late.

In the current finding, DPs were more likely to be tobacco smokers and debilitated patients from households with one room. The high probability of DP in tobacco smokers might plausibly be associated with the effect of smoking on Tb treatment outcome. A prospective cohort from Jordan reported that the risk of a poor Tb treatment outcome was much higher (70 %) in current smokers compared to never smokers [48]. Such a co-incidence of smoking and poor Tb treatment outcome might affect timely HIV care presentation. The potential of smoking to induce coughing and other symptoms consistent with tuberculosis may delay Tb diagnosis among smokers than non-smokers and this may prohibit to seek health care services as result of poor Tb prognosis that caused by delayed diagnosis [49]. Findings of our current study also support this in which the likelihood of bedridden patients to DP was higher than working patients.

For the above reasons, Tb experts declare “Clearing the smoke around the Tb/HIV Syndemic” [50]. In addition, the current finding may also call for designing and including strategies in the routine program to reduce tobacco among Tb/HIV co-infected patients [51]. In support of this, The Center for Disease Control (CDC) also calls for prompt action to incorporate anti-smoking strategies into Tb, HIV, and Tb-HIV care, and advise to apply the World Health Organization’s MPOWER [52] strategy for lessening tobacco use [51]. This is critical issue for Tb and HIV treatment and care in contributing not only for earlier presentation for HIV care but also for developing good prognosis after linking the care [50]. However, we also advised further study to explore the association between smoking and time to present for HIV care among the population.

Bedridden patients had eight times (A*OR* = 8.3, 95 % *CI*: 2.8–25.1) increased risk of DP than patients recorded as being in working status. These findings do not come as a surprise because patients who are bedridden have probably been exposed to more infections and have poorer health outcomes, a barrier that hinders early presentation for HIV care [50, 53]. It is thus plausible to suggest routine opportunistic infections and other diseases screening in patients with Tb or HIV in order to establish early and effective management strategies to reduce preventable mortalities from these conditions. In addition, HIV screening in general population, and home based HIV testing and linking to care should also be strengthened.

Tb/HIV patients from household with two or more rooms had 50 % lesser risk (A*OR* = 0.5, 95 % *CI*: 0.3–0.8) to DP than owning a single room. This demonstrated the role of adequate housing as an important enabler of effective care at steps of HIV care continuum. It is up on this intention that the U.S. Department of Housing and Urban Development (HUD) established the Housing Opportunities for Persons With AIDS (HOPWA) program and had a bold impact in the HIV Care Continuum Initiative [3]. The inadequacy in housing can lead to overcrowding that worsens Tb conditions in poor settings and this may deter HIV care use. This is supported by the current findings that significant proportion (61.3 %) of DP participants in the current study lived with more than five individuals in a single house. These factors have also been supported by several studies conducted across the globe [54–56]. This cues the need of revising intervention framework of Tb/HIV care that was focused on health sector alone. Efforts should be made to integrate and improve prevailing social determinants particularly housing in the management of HIV/Tb co-infection [29].

The study has the following limitations that should be acknowledged. Firstly, there were incomplete data and small sample size. This may have affected the precision of the estimates. Secondly, variables that could potentially have major contribution and confounding effect (e.g. HIV related stigma) were not assessed. Thirdly, due to inadequate data of desired variables, the prevalence of DP was not further described by delayed HIV testing and delayed ART initiation after early HIV testing. Fourthly, the proportion of DP is also not described by the timing of Tb diagnosis so that the effect of early or late Tb diagnosis would have been hypothesized. Fifthly, assessment of the effect of Tb treatment outcomes- default, lost to follow up, failure or cure- on time to present for HIV care is beyond the scope of the current study. Lastly, a gold standard measure of DP for resource limited countries is not yet established. As Collaboration of Observational HIV Epidemiological Research Europe (COHERE) group set definition of DP for the population of Europe [57], research groups in Africa should also set the ‘gold standard’ definition of DP for HIV care among general adult HIV positive population, HIV positive children, HIV positive mothers and Tb/HIV co-infected patients.

## Conclusions

The findings of the current study have informed that three out of five Tb/HIV co-infected patients delayed for HIV care and delayed presenters were more likely to be tobacco smokers, bedridden patients and those who were from households with single bedroom. The existence of high DP among Tb/HIV co-infected cases needs actions to reduce the aforementioned risk factors. The findings of the current study have policy and practice implications and they call for effective management strategies for Tb/HIV co-infection including improved availability of early diagnosis and improved availability of ARVs. Such study should also be followed by further research to assess barriers of DP among other vulnerable groups such as children and mothers, and other key population.

## Additional file

Additional file 1: Multilingual abstracts in the six official working languages of the United Nations. (PDF 763 kb)

## Abbreviations

AHR: Adjusted hazard ratio
AIDS: Acquired immuno deficiency syndrome
ART: Antiretroviral therapy
DP: Delayed presentation for HIV care
HIV: Human immunodeficiency virus
IRIS: Immune reconstitution inflammatory syndrome
JUTH: Jimma University Teaching Hospital
MDR: Multi drug resistance
Tb: Tuberculosis
WHO: World Health Organization
