# Supplementary material for: The prevalence and associated factors for delayed presentation for HIV care among tuberculosis/HIV co-infected patients in Southwest Ethiopia: a retrospective observational cohort
Source: Infect Dis Poverty. 2016 Nov 2;5:96. doi: 10.1186/s40249-016-0193-y (PMC5090949; doi:10.1186/s40249-016-0193-y)
Supplement: Additional file 1: — Multilingual abstracts in the six official working languages of the United Nations. (PDF 763 kb) [file 40249_2016_193_MOESM1_ESM.pdf]

Translation of the abstract into the six official working languages of the United Nations

عوامل الانتشار وما يرتبط بها لتأخر الحصول على الرعاية الصحية لفيروس نقص المناعة البشرية بين المرضى المصابين بكل من السل وفيروس نقص المناعة البشرية في جنوب غرب إثيوبيا: دراسة تعرض رصدية رجعية

هايلاي جيسيسيو، بيرتوكان تسيهاينيه، ديساليجن ماسا، أمانويل تسفاي، هافتي كاهسي، ليليان موانري

ملخص

خلفية: الحضور المتأخر للحصول على الرعاية الصحية لفيروس نقص المناعة البشرية (HIV) (أي الالتحاق المتأخر بنظام الرعاية الصحية لفيروس نقص المناعة البشرية بسبب التأخر في تشخيص الإصابة بفيروس نقص المناعة البشرية أو تأخر الالتحاق ببرنامج الرعاية الصحية لفيروس نقص المناعة البشرية بعد تشخيص الإصابة بالإيدز) هو خطوة حاسمة في سلسلة حصول مريض فيروس نقص المناعة البشرية على الرعاية المستمرة. في إثيوبيا، الحضور المتأخر للحصول على الرعاية الصحية (DP) لفيروس نقص المناعة البشرية الرعاية بين الفئات الضعيفة مثل مرضى المصابين بكل من السل وفيروس نقص المناعة البشرية المشترك لم يتم تقييمها. نحن تهدف إلى تقييم مدى انتشار والعوامل المرتبطة مع الحضور المتأخر للحصول على الرعاية الصحية (CD4<200 خلية/ميكرو لتر في الزيارة الأولى) بين المرضى المصابين بكل من السل وفيروس نقص المناعة البشرية في جنوب غرب إثيوبيا.

الأساليب: دراسة تعرض رصدية رجعية جمعت بيانات المرضى المصابين بكل من السل وفيروس نقص المناعة البشرية من المستشفى التعليمي جامعة جيما في الفترة من سبتمبر 2010 وأغسطس 2012. استخدم نموذج الانحدار اللوجستي لتحليل البيانات عند قيمة P ≥ 0.05 في النموذج النهائي.

النتائج: كان انتشار الحضور المتأخر للحصول على الرعاية الصحية بين المرضى المصابين بكل من السل وفيروس نقص المناعة البشرية 59.9٪. كان المرضى المصابون بكل من السل وفيروس نقص المناعة البشرية الذين لديهم منزل به غرفتين على الأقل أقل احتمالا (A*OR*، 0.5، 95٪ CI: 0.3-1.0) في التأخر للحضور للحصول على الرعاية الصحية عن هؤلاء الذين يملكون منازلا من غرفة واحدة فقط. كان غير مستخدمي التبغ من المشاركين المصابين بكل من السل وفيروس نقص المناعة البشرية أيضا 50٪ أقل عرضة (A*OR*، 0.5، 95٪ CI: 0.3-0.8) للتأخر في الحضور للحصول على الرعاية الصحية لفيروس نقص المناعة البشرية مقارنة مع مستخدمي التبغ. كانت الاحتمالات النسبية للحضور المتأخر للحصول على الرعاية الصحية بين المرضى المصابين بكل من السل وفيروس نقص المناعة البشرية المرضى غير الملازمين للفراش (A*OR*، 1.8، 95٪ CI، 1.0-3.1) وطريحي الفراش (A*OR*، 8.3، 95٪ CI، 2.8حتي 25،1) كان الحالة الوظيفية أعلى من حالة العمل.

الاستنتاجات: ثلاثة من أصل خمسة مرضى مصابين بكل من السل وفيروس نقص المناعة البشرية حضروا في وقت متأخر للحصول على الرعاية الصحية من فيروس نقص المناعة البشرية. وقد لوحظت نسب أعلى من الحضور المتأخر للحصول على الرعاية الصحية في المرضى طريحي الفراش، ومدخني التبغ، وأولئك الذين يقيمون من بيت من غرفة واحدة. هذه النتائج لها آثار في التدخل وتدعو إلى اتباع استراتيجيات الإدارة الفعالة في العدوى في المرضى المصابين بكل من السل وفيروس نقص المناعة البشرية بما في ذلك تشخيص فيروس نقص المناعة البشرية في وقت مبكر والانضمام في وقت مبكر للحصول على خدمات الرعاية الصحية لفيروس نقص المناعة البشرية.

Translated from English version into Arabic by Mahmoud Sami, through

TRANSLATORS
WITHOUT BORDERS

埃塞俄比亚西南部肺结核和 HIV 合并感染患者延迟就诊 HIV 的情况及其相关因素：回顾性队列研究

Hailay Gesesew, Birtukan Tsehaineh, Desalegn Massa, Amanuel Tesfay, Hafte Kahsay, Lillian Mwanri

摘要

引言：HIV 患者延迟就诊是由于其延迟进行 HIV 检测或在 HIV 诊断阳性后未及时接受相应的 HIV 治疗，这是 HIV 患者一系列治疗系统中关键的一步。在埃塞俄比亚，肺结核和 HIV 合并感染等脆弱人群的 HIV 延迟就诊情况还不清楚。本研究旨在评估埃塞俄比亚西南部肺结核和 HIV 合并感染患者（首诊的 CD4 <200 cells/µl）中延迟就诊 HIV 的情况及其相关因素。
方法：采用回顾性观察队列研究，整理 2010 年 9 月—2012 年 8 月间 Jimma 大学教学医院肺结核和 HIV 相关数据，应用 logistic 回归模型分析。
结果：59.9%的肺结核和 HIV 合并感染者延迟就诊。其中，拥有至少两个房间房子的合并感染患者比仅拥有一个房间的更少出现延迟就诊（A*OR*，0.5；95% *CI*：0.3-1.0），不抽烟的患者比抽烟的少将近 50%的延迟就诊（A*OR*，0.5；95% *CI*：0.3-0.8），能行动的患者（A*OR*，1.8；95% *CI*，1.0-3.1）和久病不起的患者（A*OR*，8.3；95% *CI*，2.8-25.1）比工作状态的患者更易延迟就诊。
结论：3/5 的肺结核和 HIV 合并感染患者延迟就诊 HIV。久病不起的患者、抽烟的患者和仅拥有一个房间的患者延迟就诊的比例更高。以上这些发现为在肺结核和 HIV 合并感染患者中开展包括 HIV 的早期诊断和早期获得 HIV 治疗在内的干预措施和有效管理措施提供启示。

Translated from English version into Chinese by Jian-hai Yin, edited by Pin Yang

CHINESE CENTER FOR DISEASE CONTROL AND PREVENTION
NATIONAL INSTITUTE OF PARASITIC DISEASES

La prévalence et les facteurs associés du retard de présentation aux soins du VIH parmi les patients atteints d'une co-infection tuberculose/VIH dans le Sud-Ouest de l'Éthiopie : une étude rétrospective par observation de cohortes

Hailay Gesesew, Birtukan Tsehaineh, Desalegn Massa, Amanuel Tesfay, Hafte Kahsay, Lillian Mwanri

Résumé
Contexte: un retard de présentation à des soins de prise en charge du virus de l'immunodéficience humaine (VIH) (il s'agit d'un recours tardif aux soins VIH en raison d'un retard du dépistage du VIH ou une intégration retardée aux soins VIH après l'obtention d'un diagnostic positif du VIH) constitue une étape critique dans la série du parcours de soins des patients atteints du VIH. En Éthiopie, le retard de présentation (RP) aux soins du VIH parmi les groupes vulnérables tels que les patients atteints d'une co-infection tuberculose (TB)/VIH n'a pas été évaluée. Nous nous sommes efforcés d'évaluer la prévalence et les facteurs associés au RP (CD4 <200 cellules/µl à la première consultation) parmi les patients atteints d'une co-infection TB/VIH dans le Sud-Ouest de l'Éthiopie.
Méthodes: une étude rétrospective par observation de cohortes a consisté à regrouper des données TB/VIH de l'Hôpital universitaire Jimma pendant une période allant de septembre 2010 à août 2012. L'analyse de données a consisté à utiliser un modèle de régression logistique à une valeur P de≤0,05 dans le modèle final.
Résultats: la prévalence du RP parmi les patients atteints d'une co-infection TB/VIH s'élevait à 59,9 %. Les patients atteints d'une co-infection TB/VIH occupant une maison composée d'au moins deux pièces avaient moins de chance (A*OR*, 0,5; *IC* à 95%: 0,3-1,0) de se présenter en retard par rapport à ceux ne disposant que d'une seule pièce. Les non-fumeurs parmi les patients atteints d'une co-infection TB/VIH présentaient aussi 50% de moins de chance (A*OR*, 0,5; *IC* à 95%: 0,3-0,8) de se présenter en retard à des soins VIH par rapport aux fumeurs. Les probabilité relatives du RP parmi les patients atteints d'une co-infection TB/VIH affichant un statut fonctionnel ambulatoire (A*OR*, 1,8; *IC* à

95%, 1,0-3,1) et alité (A*OR*, 8,3; *IC* à 9 %, 2,8-25,1) étaient supérieures à celles des patients en état de travailler.
Conclusions: trois patients atteints d’une co-infection TB/VIH sur cinq se sont présentés en retard aux soins VIH. Des proportions supérieures de RP étaient observées chez les patients alités, les fumeurs et ceux dont le logement se composait d’une seule pièce. Ces résultats ont des implications pour l’intervention et soulignent la nécessité de mise en œuvre de stratégies efficaces de prise en charge de la co-infection TB/VIH, y compris le diagnostic précoce du VIH et l’intégration précoce à des services de prise en charge du VIH.

Translated from English version into French by eric ragu, through

Распространенность и связанные с ней факторы поздней госпитализации при превенции ВИЧ среди больными среди пациентов с сочетанной инфекцией туберкулез/ВИЧ на юго-западе Эфиопии: ретроспективное наблюдение за группой

Хайлай Гесесев, Биртукан Цехайне, Деселен Масса, Амануэль Тесфай, Халфе Кахсай, Лилиан Мванри

Резюме
Подоплека: Поздняя госпитализация пациентов с вирусом иммунодефицита человека (ВИЧ) (позднее привлечение к лечению ВИЧ из-за задержки с тестированием на ВИЧ или несвоевременным обращением в связи с ВИЧ после положительного диагноза ВИЧ) является критическим процессом в уходе за пациентами с ВИЧ. В Эфиопии поздняя госпитализация (DP) пациентов с ВИЧ среди уязвимых групп, таких как больных туберкулезом (ТБ) / с сочетанной инфекцией ВИЧ, не оценивалась. Нашей целью было оценить распространенность и факторы, связанные с DP (CD4 <200 клеток/мкл при первом визите) среди пациентов с сочетанной инфекцией ТБ / ВИЧ в юго-западной части Эфиопии.
Методы: Ретроспективное наблюдение за группой сопоставляется с данными по ТБ / ВИЧ из клинической больницы Джиммы за период с сентября 2010 года по август 2012 года. Для анализа данных использовалась регрессионная логистическая модель при значении $P \leqslant 0{,}05$ итоговой модели.
Результаты: Распространенность DP среди пациентов с сочетанной инфекцией ТБ/ВИЧ составила 59,9%. ТБ / ВИЧ-инфицированных пациентов, у которых был дом минимум с двумя комнатами, была менее вероятна (AOR, 0,5; 95% *CI*: 0,3-1,0), чем среди тех, у которых была только одна комната. Неупотребляющие табак пациенты ТБ / ВИЧ на 50% реже (AOR, 0,5; 95% ДИ: 0,3-0,8) госпитализировались поздно, по сравнению с курильщиками. Относительные шансы DP среди пациентов с сочетанной инфекцией ТБ/ВИЧ с амбулаторными (AOR, 1,8; 95% *CI*: 1.0-3.1) и прикованными к постели (AOR, 8,3; 95% *CI*: 2.8-25.1), их функциональный статус был выше, чем рабочий.

Заключение: Три из пяти пациентов с сочетанной инфекцией ТБ / ВИЧ были поздно госпитализированы для лечения ВИЧ. Более высокие доли DP наблюдались среди лежачих больных, курильщиков табака, а также тех, кто проживал в однокомнатной квартире. Эти выводы были получены экспериментальным путем и требуют эффективной стратегии управления по борьбе с туберкулезом / ВИЧ-инфекцией, включая раннюю диагностику ВИЧ-инфекции, и требуют привязывания к услугам по уходу за ВИЧ.

Translated from English version into Russian by Jekaterina Merkuljeva, through

La prevalencia y los factores asociados a las visitas tardías para recibir atención médica por VIH entre los pacientes coinfectados por VIH y tuberculosis en el sudoeste de Etiopía: un estudio observacional retrospectivo de cohortes

Hailay Gesesew, Birtukan Tsehaineh, Desalegn Massa, Amanuel Tesfay, Hafte Kahsay, Lillian Mwanri

Resumen

Antecedentes: La cuestión de las visitas tardías de los pacientes con virus de la inmunodeficiencia humana (VIH) para recibir atención médica, es decir, un tratamiento diferido del VIH debido a un retraso en la realización de la prueba del VIH o un retraso en el tratamiento tras una prueba positiva de VIH, es un componente crítico de la continuidad asistencial en la serie de pacientes con VIH. En Etiopía, no se ha evaluado la repercusión de las visitas tardías al médico entre las personas con VIH de grupos vulnerables, como los pacientes coinfectados por VIH y tuberculosis (Tb). Nuestro objetivo fue el de evaluar la prevalencia de los factores asociados a las visitas tardías (CD4 < 200 células/μl en la primera visita) entre los pacientes coinfectados por VIH y Tb en el sudoeste de Etiopía.

Métodos: En un estudio observacional retrospectivo de cohortes, se recopilaron datos de pacientes coinfectados por VIH y Tb del Hospital Universitario de Jimma, pertenecientes al período de septiembre de 2010 a agosto de 2012. Para el análisis de los datos se utilizó un modelo de regresión logística con un valor de $p \leqslant 0{,}05$ en el modelo final.

Resultados: La prevalencia de visitas tardías al médico entre los pacientes coinfectados por VIH y Tb fue del 59,9%. Entre los pacientes coinfectados por VIH y Tb cuya vivienda tenía al menos dos habitaciones, las visitas tardías al médico se daban en menor grado (*razón de posibilidades ajustada* (A*OR)*, 0,5; 95% *CI*: 0,3-1,0) que entre los pacientes cuya vivienda era de una sola habitación. Los pacientes coinfectados por VIH y Tb no fumadores también presentaban una tendencia menor a acudir tarde al médico, un 50 % menor (A*OR*, 0,5; 95% *CI*: 0,3-0,8) que los fumadores. La razón de posibilidades (odds ratio) de visitas tardías entre los pacientes coinfectados por VIH y Tb con un estado funcional deficiente, tanto los que podían levantarse (A*OR*, 1,8; 95% *CI*, 1,0-3,1) como los encamados (A*OR*, 8,3; 95% *CI*, 2,8-25,1), era más elevada que entre los pacientes con un estado funcional normal.

Conclusiones: De cada cinco pacientes coinfectados por VIH y Tb, tres acudieron tarde para recibir tratamiento contra el VIH. Se observó una proporción más elevada de visitas tardías entre los pacientes encamados, los fumadores y aquellos cuya vivienda era de una sola habitación. Estas observaciones

tienen repercusiones en lo que respecta a las intervenciones y ponen de relieve la necesidad de unas estrategias efectivas de tratamiento de la coinfección por VIH y Tb, como el diagnóstico precoz de VIH y el tratamiento poco después del diagnóstico.

Translated from English version into Spanish by Mar Jiménez Quesada, through

TRANSLATORS
WITHOUT BORDERS
